# Data on respiratory variables in critically ill patients with acute respiratory failure placed on proportional assist ventilation with load adjustable gain factors (PAV+)

**DOI:** 10.1016/j.dib.2016.05.078

**Published:** 2016-06-07

**Authors:** Dimitris Georgopoulos, Nectaria Xirouchaki, Nikolaos Tzanakis, Magdy Younes

**Affiliations:** a Intensive Care Medicine and University Hospital of Heraklion, Medical School, University of Crete, Heraklion, Crete, Greece; b Pulmonary Department, University Hospital of Heraklion, Medical School, University of Crete, Heraklion, Crete, Greece; c Sleep Disorders Centre, Misericordia Health Centre, Winnipeg, Manitoba, Canada

**Keywords:** PAV+, Proportional assist ventilation with load adjustable gain factors, CMV, Controlled mechanical ventilation (Passive mechanical ventilation), Rmin, End-inspiratory airway resistance during controlled mechanical ventilation, PEEPi, Intrinsic positive end-expiratory airway pressure, *V_T_*, Tidal volume, Crs, Respiratory system compliance, Δ*P*, Driving pressure, VT_CMV_, Tidal volume during controlled mechanical ventilation, Crs_CMV_, Respiratory system compliance during controlled mechanical ventilation, Δ*P*_CMV_, Driving pressure during controlled mechanical ventilation, VT_PAV+aver_, Average tidal volume during the first 8-h period of proportional assist ventilation with load adjustable gain factors, Crs_PAV+aver_, Average respiratory system compliance during the first 8-hour period of proportional assist ventilation with load adjustable gain factors, Δ*P*_PAV+aver_, Average driving pressure during the first 8-h period of proportional assist ventilation with load adjustable gain factors, VT_PAV+_, Tidal volume during proportional assist ventilation with load adjustable gain factors, Crs_PAV+_, Respiratory system compliance during proportional assist ventilation with load adjustable gain factors, Δ*P*_PAV+_, Driving pressure during proportional assist ventilation with load adjustable gain factors, PaCO_2_, Partial pressure of arterial CO_2_, ARDS, Acute respiratory distress syndrome, Tidal volume, Compliance, Driving pressure

## Abstract

The data show respiratory variables in 108 critically ill patients with acute respiratory failure placed on proportional assist ventilation with load adjustable gain factors (PAV+) after at least 36 h on passive mechanical ventilation. PAV+ was continued for 48 h until the patients met pre-defined criteria either for switching to controlled modes or for breathing without ventilator assistance. Data during passive mechanical ventilation and during PAV+ are reported. Data are acquired from the whole population, as well as from patients with and without acute respiratory distress syndrome. The reported variables are tidal volume, driving pressure (Δ*P*, the difference between static end-inspiratory plateau pressure and positive end-expiratory airway pressure), respiratory system compliance and resistance, and arterial blood gasses. The data are supplemental to our original research article, which described individual Δ*P* in these patients and examined how it related to Δ*P* when the same patients were ventilated with passive mechanical ventilation using the currently accepted lung-protective strategy “Driving pressure during assisted mechanical ventilation. Is it controlled by patient brain?” [1].

Specifications Table

**Table:** 

Subject area	Biology
More specific subject area	Control of breathing
Type of data	Table, Figures
How data was acquired	Ventilator monitor (Puritan Bennett 840, Nellcor Puritan Bennett LLC, Gosport, UK), blood gas analyzer (Roch, Germany)
Data format	Individual, analyzed
Experimental factors	PAV+
Experimental features	Critically ill patients with acute respiratory failure were placed on PAV+ after at least 36 h on passive mechanical ventilation (CMV). PAV+ was continued for 48 h until the patients met pre-defined criteria either for switching to controlled modes or for breathing without ventilator assistance. During PAV+ and CMV various respiratory variables were measured.
Data source location	Intensive Care Unit, University Hospital of Heraklion, Medical School, University of Crete, Heraklion, Crete, Greece.
Data accessibility	Data is within the article

Value of the data

• *Individual respiratory variables including driving pressure in mechanically ventilated critically ill patients during assisted mechanical ventilation using a mode that does not restrict breathing pattern are reported.*
• *May stimulate further research in critically ill patients on the ability of the feedback systems of regulation of breathing to protect the lungs from ventilator induced lung injury.*
• *May facilitate new approaches for titrating ventilator settings in critically ill patients.*

## 1 Data

The data show *V_T_*, Crs and Δ*P* in critically ill patients during PAV+ and CMV, and the changes in these variables when patients were switched from CMV to PAV+. Data in patients with and without ARDS are presented, as well as Rmin and PEEPi during CMV in these patients. The relationship between PaCO_2_ and Δ*P* during PAV+ is also shown (Figs. 1–11).

## 2 Experimental design, materials and methods

### 2.1 Patients

Patients under mechanical ventilation for at least 36 h and ventilated with a controlled mode (CMV, volume or pressure control) were screened for eligibility. Enrollment criteria required absence of the following [2]: a do-not-resuscitate order, mechanical ventilation with assisted modes (independent of the duration), expected poor short-term prognosis (<3 months), neuromuscular disease with respiratory muscle involvement that could permanently impair the ability to breathe spontaneously, and age <18 and >85 years. Inclusion criteria were the ability to trigger the ventilator at a satisfactory rate (>10 breaths/min); PaO_2_>60 mmHg, with fractional concentration of inspired O_2_ (FIO_2_) of <65%; total [extrinsic (PEEP) and intrinsic (PEEPi)] positive end-expiratory airway pressure (PEEP_TOT_=PEEP+PEEPi)<15 cmH_2_O; no severe acidemia (pH>7.30); no severe hemodynamic instability defined as a need for norepinephrine infusion at a rate greater than 0.5 μg/kg/h; no severe bronchospasm [end-inspiratory airway resistance (Rmin) measured during CMV <20 cmH_2_O/l/sec], and a stable neurological status. Stable neurological status was defined as no need for: (1) heavy sedation to control intracranial pressure and (2) any intervention during the previous 24 h either to lower intracranial pressure to normal values (≤12 cmH_2_O) or to manage any event related to the central nervous system (i.e. seizures). The ability to trigger the ventilator was evaluated by placing the patients on Bilevel Positive Airway Pressure (BIPAP) or assist-volume control mode, and adjusting the ventilator rate downward (Table 1).

### 2.2 Measurements during CMV and PAV+

During CMV and the 48-h PAV+ period, the following parameters were measured at specific time intervals.

1) Gas exchange data: PaO_2_, PaCO_2_, PaO_2_/F_I_O_2_, and pH
2) Respiratory data: *V_T_* (calculated as the ratio of minute ventilation to ventilator rate, which were measured by averaging data over 1 min), end-inspiratory alveolar pressure during CMV and PAV+ (*P*plat_CMV_, *P*plat_PAV+_, respectively), respiratory system compliance (Crs_CMV_, Crs_PAV+_), PEEP, PEEPi and PEEP_TOT_ (see below).

### 2.3 Respiratory system mechanics during controlled mechanical ventilation (CMV)

During CMV, respiratory system mechanics were assessed within 8 h before switching to PAV+ (when criteria for passive ventilation were met). If the patients were ventilated on volume control mode, respiratory system mechanics were measured at settings in which the patients had previously been ventilated. If the patients were ventilated on pressure control mode, respiratory system mechanics were measured by placing them on volume control. The ventilator rate remained constant and the ventilator was set to deliver a *V_T_* similar to that achieved with the pressure control. A square wave inspiratory flow-time profile was used. The mechanical properties of the respiratory system were determined using the occlusion technique [3], [4]. Briefly, the airways were occluded at end-inspiration for 3 s; there was an immediate drop in airway pressure from a peak (*P*peak) to a lower value (*P*_1_), followed by a gradual decay to a plateau (*P*plat). In each patient, at least 3 breaths with satisfactory plateau were analyzed and the mean values were reported. Intrinsic PEEP (PEEPi) was measured by occluding the airways at the end of a tidal expiration for 3 seconds and observing the airway pressure. Again, 3 breaths were analyzed. Respiratory system static inflation end-inspiratory compliance (Crs_CMV_), end-inspiratory airway resistance (*R*_min_, the “ohmic” component of airway resistance), end-inspiratory total resistance (*R*_max_) and the resistance due to time constant inequalities and/or viscolelastic properties (Δ*R*=*R*_max_−*R*_min_) of respiratory system were computed according to standard formulas [3], [4]. The endotracheal tube resistance was not taken into account.

### 2.4 Estimation of respiratory system compliance during PAV+ (Crs_PAV+_)

A software program is built into the ventilator which, when proportional assist ventilation mode (PAV+) is activated, estimates the compliance (Crs_PAV+_) of respiratory system, based on methods previously described [5]. Briefly, at random intervals of 4–10 breaths, a 300 ms pause maneuver at the end of inspiration is applied and the Paw at end-inspiratory pause time (Pplat_PAV+_) is measured. Given that Pplat_PAV+_ (1) is equal to end-inspiratory alveolar pressure (Palv) and (2) during the interval of obstruction inspiratory muscle pressure returns to zero [5], Crs_PAV+_ is calculated as follows:

(1) $${\mathrm{Crs}}_{\text{PAV}+}=\text{VT}/\left({\mathrm{Pplat}}_{\text{PAV}+}{-\text{PEEP}}_{\mathrm{TOT}}\right){/V}_{T},$$

PEEPi is estimated by the ventilator software using the following technique [2]. Since Crs_PAV+_ has been measured, the software, assuming that expiration is passive, estimates Palv continuously from the beginning to the end of expiration. If expiratory flow continues until shortly before the next trigger, PEEPi is calculated as the difference between estimated Palv and PEEP 100 ms before the next trigger. If expiratory flow becomes zero before a breath is triggered, then Palv=Paw=PEEP and thus Palv–PEEP=0 (i.e. PEEPi=0).

In an automated system in which interventions are applied randomly under unsupervised conditions, safeguards need to be included to ensure that data obtained under unfavorable conditions are filtered out. Thus, all raw data are subjected to checks, and the estimates of Crs_PAV+_ are discarded if any of the rejection pre-defined criteria are met [5]. Valid estimates of Crs_PAV+_ are required for breath delivery, and are constantly updated by averaging new values with previous values. If new values for Crs_PAV+_ are rejected, the previous values remain active until valid new values are obtained. The ventilator software monitors the update process and generates an escalating alarm condition if the old values do not refresh.

The driving pressure during PAV+ (Δ*P*_PAV+_) is calculated as VT/Crs_PAV+_. Δ*P*_PAV+_ is calculated with and without taking PEEPi into consideration. Crs_PAV+_ without taking PEEPi into consideration, is estimated as follows:

(2) $${\mathrm{Crs}}_{\text{PAV}+}=\text{VT}/\left({\mathrm{Pplat}}_{\text{PAV}+}-\text{PEEP}\right){/V}_{T},$$

It follows that Δ*P*_PAV+_ without taking PEEPi into consideration, is the difference between Pplat_PAV+_−PEEP.

### 2.5 Statistical analysis

Data are given as median (25th–75th interquartile range), unless stated otherwise. Proportions were compared using the Fisher exact test. Continuous variables were compared with Wilcoxon and Man–Whitney tests, as appropriate. Regression analysis was performed using the least square method. Linear mixed effect models on parameters of repeated measurements were used to investigate changes in various variables over time during PAV+. The values of the first four serial measurements, corresponding to an 8-h PAV+ period, were included in the model in order to compare with the corresponding variables obtained within the 8-h CMV period. *P*<0.05 was considered as significant.

## Figures and Tables

**Fig. 1:**
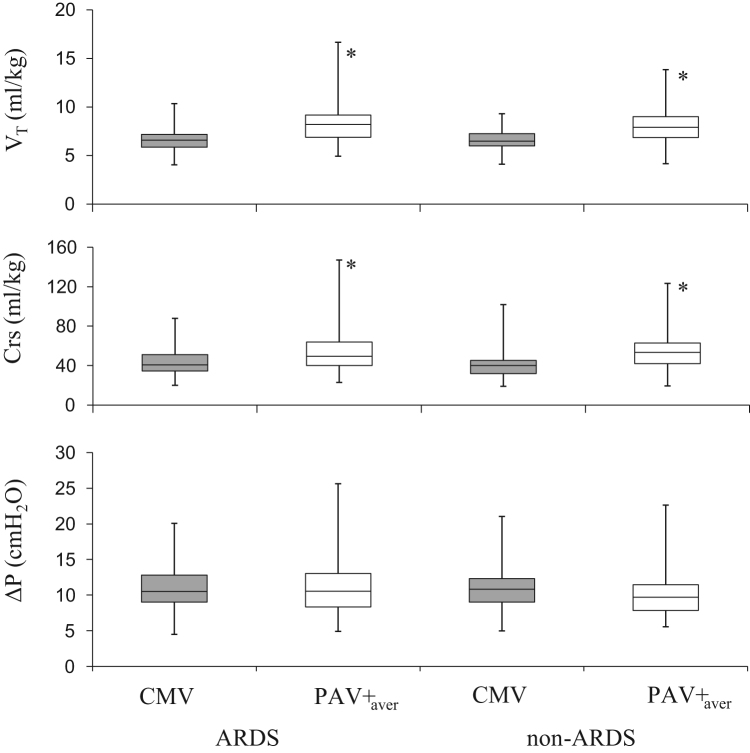
Boxplot with whiskers from minimum to maximum of *V_T_*, Crs and Δ*P* in ARDS (*n*=64) and non-ARDS patients (*n*=44) during CMV (grey box) and PAV+. Data during PAV+ were averaged (open box, PAV+_aver_) during the first 8-h PAV+ period (time 0, 1, 4 and 8 h). *Significantly different from CMV (*p*<0.0001).

**Fig. 2:**
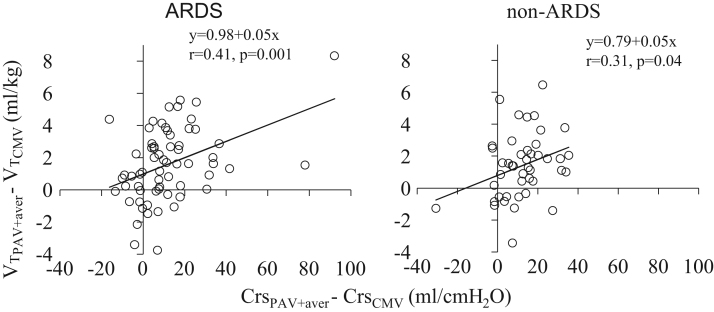
Individual relationships between the change in *V_T_* and that of Crs when the patients were switched from CMV to PAV+ in ARDS (*n*=64) and non-ARDS (*n*=44) patients. Continuous lines; Regression lines. During CMV, the measurements of *V_T_* and Crs were obtained within 8 h before switching to PAV+ when criteria for passive mechanical ventilation were met (VT_CMV_, Crs_CMV_). *V_T_* and Crs during PAV+ were obtained by averaging these variables during the first 8-h PAV+ period (VT_aver_, Crs_PAV+aver_). Therefore, each patient was characterized by a single data point. The percentage of patients in whom VT_PAV+aver_ increased while Crs_PAV+aver_ decreased did not differ between ARDS and non-ARDS patients (15.6% vs. 6.8%, *p*=0.23). VT_PAV+aver_−VT_CMV_; difference in tidal volume between PAV+ (average data) and CMV. Crs_PAV+aver_−Crs_CMV_; difference in respiratory system compliance between PAV+ (average data) and CMV.

**Fig. 3:**
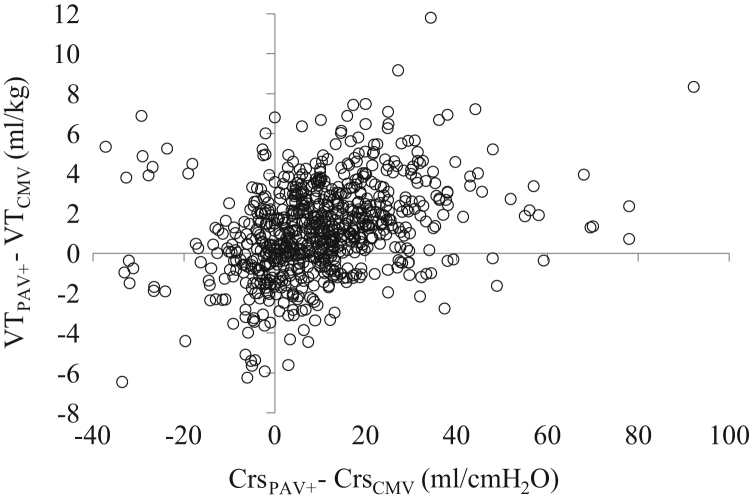
Individual relationships (all patients) between the change in *V_T_* (difference in *V_T_* between PAV+ and CMV, VT_PAV+_−VT_CMV_) and that of Crs (difference in Crs between PAV+ and CMV, Crs_PAV+_−Crs_CMV_) when the patients were switched from CMV to PAV+. All measurements (*n*=744). In each patient during CMV, one measurement was performed, while during PAV+, multiple measurements at different time points were obtained [8 (4–10) measurements per patient, median (interquartile range)]. Therefore, each patient was characterized by a number of data points equal to the number of measurements during PAV+.

**Fig. 4:**
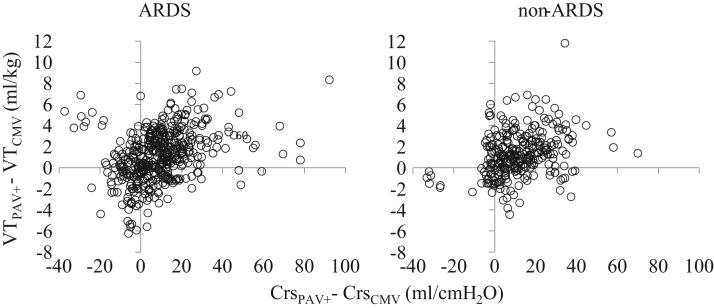
Individual relationships in ARDS and non-ARDS patients between the change in *V_T_* (difference in *V_T_* between PAV+ and CMV, VT_PAV+_−VT_CMV_) and that of Crs (difference in Crs between PAV+ and CMV, CrsP_PAV+_−Crs_CMV_) when the patients were switched from CMV to PAV+. ARDS: 64 patients, 457 measurements, left. Non-ARDS: 44 patients, 287 measurements, right. Each patient was characterized by a number of data points equal to the number of measurements during PAV+ (see also Fig. 3). The percentage of measurements in which *V_T_* increased (compared to CMV) while Crs_PAV+_ decreased (compared to CMV) did not differ between ARDS and non-ARDS patients (10.3% vs. 7.3%, *p*=0.19).

**Fig. 5:**
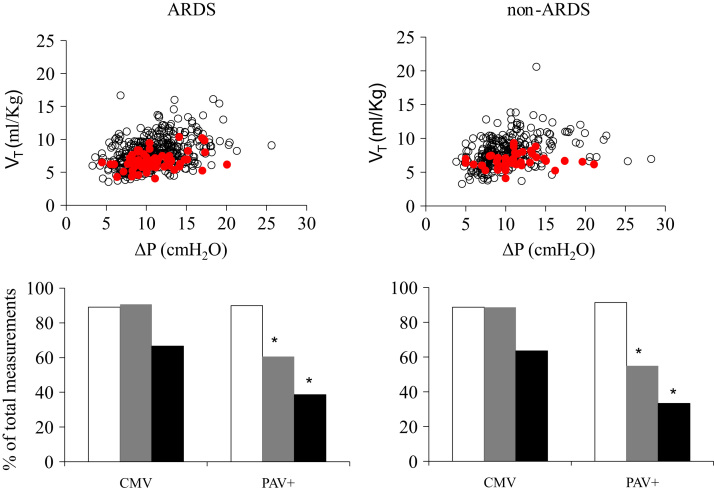
Upper panel: Individual *V_T_*–ΔP relationships in ARDS and non-ARDS patients during PAV+ (open black circles) and CMV (closed, red circles). While during CMV each patient was characterized by a single data point, during PAV+ each patient was characterized by multiple data points (depending on the number of measurements during PAV+). Lower panel: Percentage of total measurements in which Δ*P* was <15 cmH_2_O (open bars) and *V_T_* was <7 ml/kg (black bars) and <8 ml/kg (gray bars) during CMV and PAV+. Left: ARDS (64 patients, 457 measurements during PAV+). Right: non-ARDS (44 patients, 287 measurements during PAV+). *Significantly different from the corresponding value during CMV (*p*<0.0001). (For interpretation of the references to color in this figure legend, the reader is referred to the web version of this article.)

**Fig. 6:**
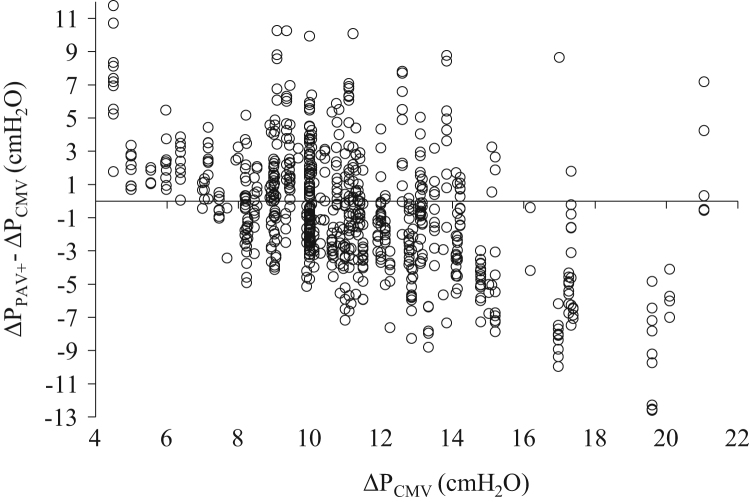
Individual changes (all patients) in Δ*P* when the patients were switched from CMV to PAV+ (Δ*P*_PAV+_−Δ*P*_CMV_) as a function of Δ*P*_CMV_. All measurements (*n*=744). Each patient was characterized by a number of data points equal to the number of measurements during PAV+. Notice that when Δ*P*_CMV_ was ≤8 cmH_2_O the patients increased Δ*P*_PAV+_ in 59 out of 65 measurements (90.8%), while when Δ*P*_CMV_ was ≥15 cmH_2_O the patients decreased Δ*P*_PAV+_ in 58 out of 67 measurements (86.6%).

**Fig. 7:**
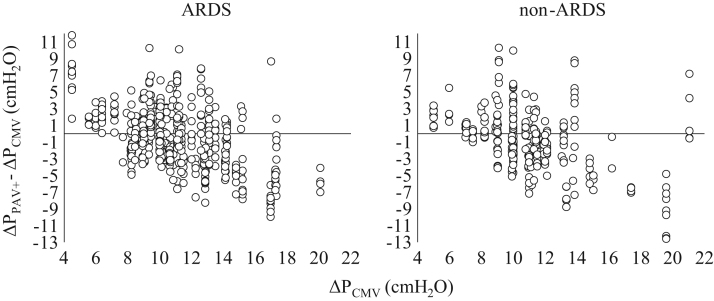
Individual changes in Δ*P* in ARDS and non-ARDS patients when the patients were switched from CMV to PAV+ (Δ*P*_PAV+_−Δ*P*_CMV_) as a function of Δ*P*_CMV_. Each patient was characterized by a number of data points equal to the number of measurements during PAV+. Notice that when Δ*P*_CMV_ in ARDS patients were ≤8 cmH_2_O, they increased Δ*P*_PAV+_ in 38 out of 41 measurements (92.7%), while when Δ*P*_CMV_ was ≥15 cmH2O, these patients decreased Δ*P*_PAV+_ in 39 out of 45 measurements (86.7%). The corresponding values in non-ARDS patients were 21 out of 24 (87.5%, *p*=0.66) and 19 out of 22 (86.3%, *p*=1.00).

**Fig. 8:**
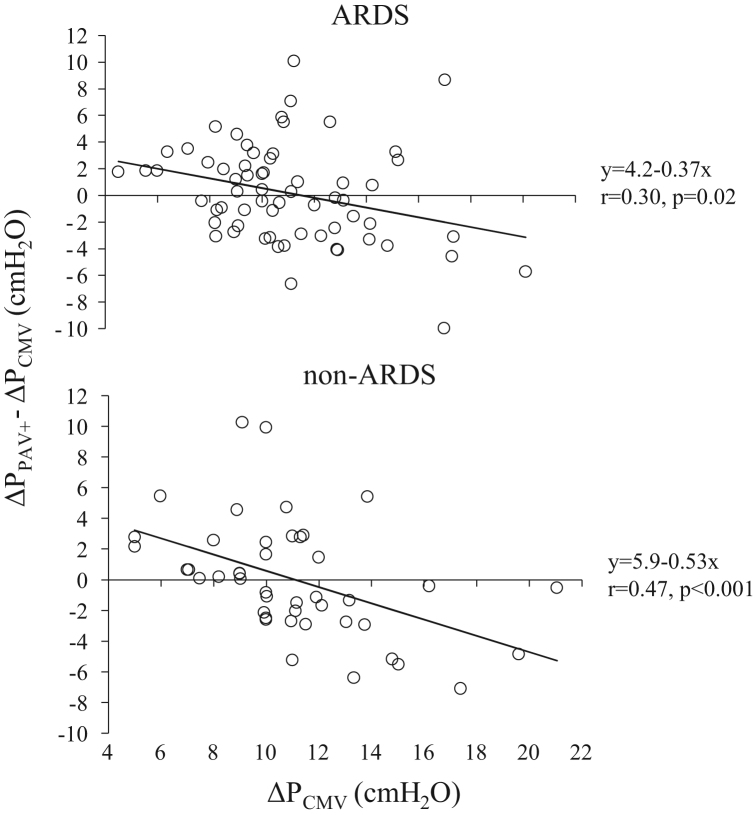
Individual changes (at 5–10 min after switching to PAV+) in Δ*P* in ARDS and non-ARDS patients when the patients were switched from CMV to PAV+ (Δ*P*_PAV+_−Δ*P*_CMV_) as a function of Δ*P*_CMV_. Continuous lines; regression lines. Each patient was characterized by a single data point.

**Fig. 9:**
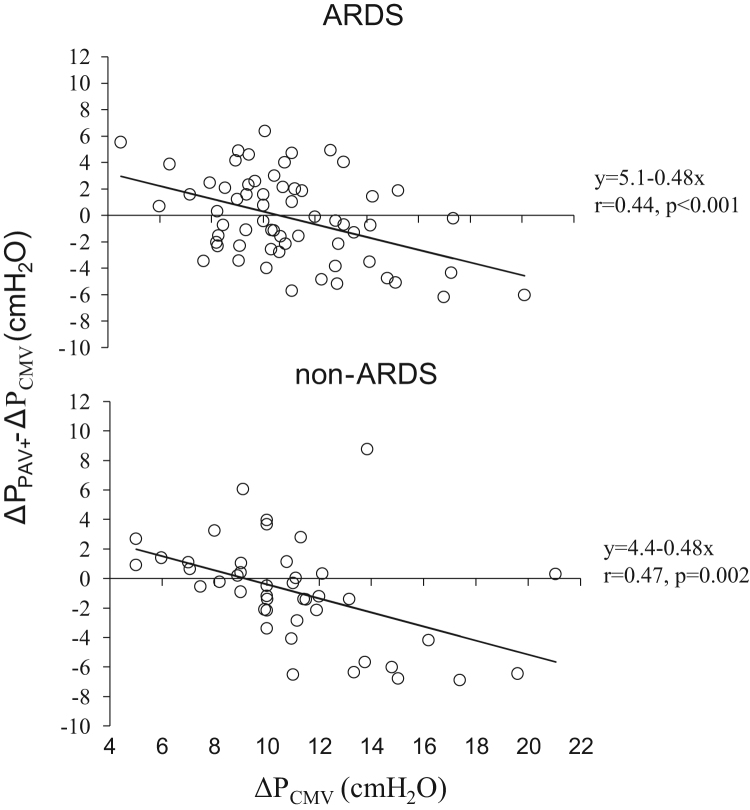
Individual changes (at 1 h after switching to PAV+) in Δ*P* in ARDS and non-ARDS patients when the patients were switched from CMV to PAV+ (Δ*P*_PAV+_−Δ*P*_CMV_) as a function of Δ*P*_CMV_. Continuous lines; regression lines. Each patient was characterized by a single data point.

**Fig. 10:**
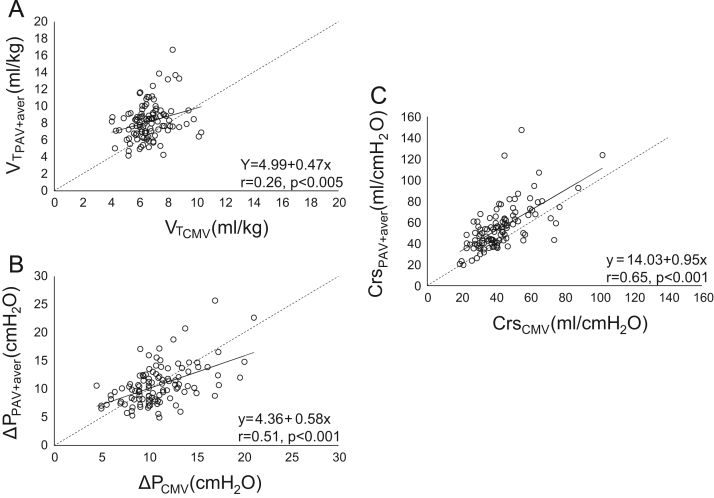
Individual relationships (all patients) between respiratory variables (VT, Crs and Δ*P*) during PAV+ (average data) and CMV (VT_CMV_ Crs_CMV_ Δ*P*_CMV_, respectively). Relationships between VT_PAV+aver_ and VT_CMV_ (A), Δ*P*_PAV+aver_ and Δ*P*_CMV_ (B), and Crs_PAV+aver_ and Crs_CMV_ (C). Continuous lines; regression lines. Dashed line; lines of identity. Significant linear relationships were observed between VT_CMV_ and VT_PAV+aver_, Δ*P*_CMV_ and Δ*P*_PAV+aver_, and Crs_CMV_ and Crs_PAV+aver_. In a given plot each patient was characterized by a single data point.

**Fig. 11:**
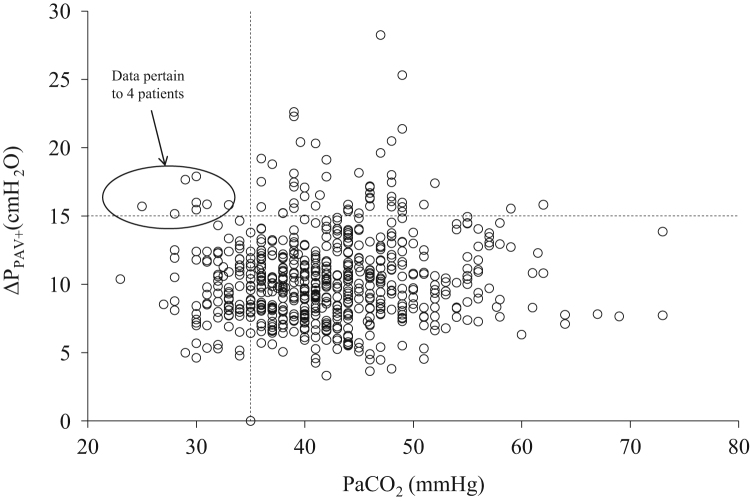
Individual relationships (all patients) between Δ*P*_PAV+_ and PaCO_2_ during PAV+. All measurements (*n*=636). Dashed horizontal and vertical lines indicate Δ*P*_PAV+_ 15 cmH_2_O and PaCO_2_ 35 mmHg, respectively. Only in 1.3% of measurements (*n*=8) hyperventilation to PaCO_2_<35 mmHg were associated with Δ*P*_PAV+_≥15 cmH_2_O. Each patient was characterized by a number of data points equal to the number of arterial blood gasses measurements during PAV+.

**Table 1.** Rmin and PEEPi in ARDS and non-ARDS patients during CMV.

	ARDS	non-ARDS
R_min_ (cmH_2_O/L/s)	10 (8–13)	10 (8–14)
PEEPi (cmH_2_O)	0 (0–1)	0 (0–1)

Values are median (interquartile range).
