# Supplementary material for: Data on respiratory variables in critically ill patients with acute respiratory failure placed on proportional assist ventilation with load adjustable gain factors (PAV+)
Source: Data Brief. 2016 Jun 7;8:484–93. doi: 10.1016/j.dib.2016.05.078 (PMC4915951; doi:10.1016/j.dib.2016.05.078)
Supplement: Supplementary file 1 — Supplementary material [file mmc1.docx]

Funding: None.

Conflict of interest: Dimitris Georgopoulos received lecture fee (honoraria) from Covidien. Magdy Younes has a patent for method of determining elastance during PAV with royalties paid, and a PAV trademark with royalties paid. The other authors declare no competing interests. Covidien was not involved in any aspect of the design or conduct of the study, the data analysis, or the manuscript preparation and presentation.
